# Health Tract, No. 303

**Published:** 1867-09

**Authors:** 


### HEALTH TRACT, No. 303.

### Illusive Memories.

"I HAVE come to know whether you think I am deranged or not," said a young lady one day, with a directness, frankness, and unembarressment very unusual. After an hour's investigation it was demonstrated that she was hopelessly deranged. The form of the malady was that when she heard an impressive sermon, an exciting address, or read an interesting article in prose or verse, she became possessed with the idea that she herself was the author and that by some unfair means others had become possessed of her own ideas.

It is yet in the memory of some, how earnestly an estimable young lady claimed a few years ago, that William Allen Butler had found her lost composition, dropped in a stage, and made out of it the celebrated article about Miss Flora McFlimsey of Madison Square, who never had anything to wear. There is at this time a very exciting controversy between a gentleman and lady, and their multitudinous friends, as to the authorship of that much admired piece of poetry, "Rock me to Sleep, Mother." Taking it for granted that the lady and gentleman are equally honest and in earnest, may it not be, as to one of them, a case of illusive memory?

Not a few readers, the writer certainly does, may remember that dreams sometime come over them as if they were a continuation of some former dream, as if the beginning of it had some connection with a previous dream. In precisely the same way the present reality may be connected with a dim, shadowy impression of a previous existence, when there had been no previous existence. We are all familiar with the fact, that a person with whom we are conversing may give an expression to a sentiment which strikes us with peculiar force, from the fact that it had been floating about in our mind's vision, but had never formed itself into definite words or phrase and we felt delighted in thus having it framed to us. It may be that a higher degree of this thing may give rise to states of the mind in relation to the authorship in question, which are really diseased or illusive memories, somewhat akin to the habit sometimes met with in persons who in the excitement of conversation will state facts, what for their purposes they wish to be facts, or as likely as not, in their view, to be facts, thus misleading others and often putting themselves in painful or humiliating positions. Tendencies of this kind should be resolutely striven against, for they grow by yielding to them, and in point of morals there is all the criminality of a deliberate falsehood. Conscientious men, men of truth and of extensive learning, are the last men in the world to be positive of anything which is not akin to demonstration; and least of all positive of anything which leans on memory alone. The weakest-minded and the least informed are habitually the most positive. The lesson is, adore Truth in writing, in conversation and in action; do not assert as a truth, which can not be demonstrated or corroborated; if need be, by outside evidence. A true gentleman habitually avoids making positive statements, thus securing himself from the charge of falsehood, and society would be greatly blessed if, in addition, the habit were sedulously cultivated of making no statement which was not literally true, with a wide margin; and if "often" and "very" were our highest expletives instead of always, never, awful, and the like.

